# Microelectromechanical Systems (MEMS) for Biomedical Applications

**DOI:** 10.3390/mi13020164

**Published:** 2022-01-22

**Authors:** Cristina Chircov, Alexandru Mihai Grumezescu

**Affiliations:** 1 Department of Science and Engineering of Oxide Materials and Nanomaterials, University Politehnica of Bucharest, 011061 Bucharest, Romania; cristina.chircov@yahoo.com; 2 National Research Center for Micro and Nanomaterials, University Politehnica of Bucharest, 060042 Bucharest, Romania; 3 Research Institute of the University of Bucharest—ICUB, University of Bucharest, 050657 Bucharest, Romania; 4 Academy of Romanian Scientists, 3 Ilfov Street, 050044 Bucharest, Romania

**Keywords:** MEMS, BioMEMS, lab-on-chip devices, microfluidics, microfabrication, diagnostics, drug delivery systems, microsurgery

## Abstract

The significant advancements within the electronics miniaturization field have shifted the scientific interest towards a new class of precision devices, namely microelectromechanical systems (MEMS). Specifically, MEMS refers to microscaled precision devices generally produced through micromachining techniques that combine mechanical and electrical components for fulfilling tasks normally carried out by macroscopic systems. Although their presence is found throughout all the aspects of daily life, recent years have witnessed countless research works involving the application of MEMS within the biomedical field, especially in drug synthesis and delivery, microsurgery, microtherapy, diagnostics and prevention, artificial organs, genome synthesis and sequencing, and cell manipulation and characterization. Their tremendous potential resides in the advantages offered by their reduced size, including ease of integration, lightweight, low power consumption, high resonance frequency, the possibility of integration with electrical or electronic circuits, reduced fabrication costs due to high mass production, and high accuracy, sensitivity, and throughput. In this context, this paper aims to provide an overview of MEMS technology by describing the main materials and fabrication techniques for manufacturing purposes and their most common biomedical applications, which have evolved in the past years.

## 1. Introduction

The tremendous advancements in electronics miniaturization have led to the birth of a novel class of devices, namely microelectromechanical systems (MEMS) [1]. Originated from the United States, the MEMS acronym can also be referred to as Microsystems Technology in Europe and Micro Machines in Japan [2]. Precisely, the term MEMS refers to micrometer-scaled precision devices, which combine mechanical and electrical components to accomplish tasks that are normally carried out by macroscopic systems [2,3,4,5,6]. With a size ranging from several micrometers to several millimeters, MEMS devices are generally produced through micromachining techniques that have originated in the integrated circuit industry, with silicon being the main material for their manufacture [1,3,4,5].

**Figure 1:**
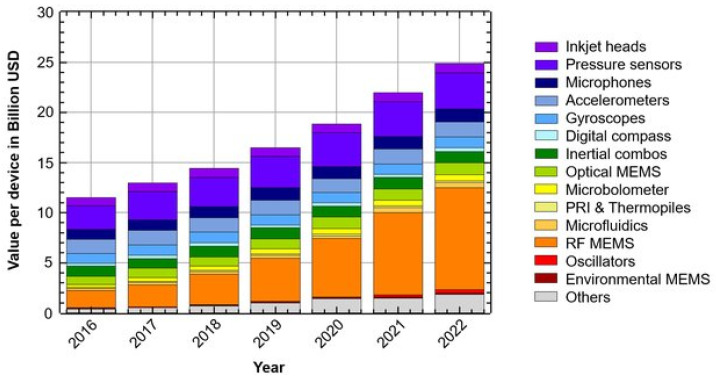
MEMS market value forecast in billion US dollars by year. Reprinted from an open-access source [43].

Considering that in 1965 the IBM360 computer filled two large rooms, the level of miniaturization that we are witnessing today due to the tremendous realizations in the semiconductor and integrated circuit technologies is overwhelming [7]. Originally developed in the microelectronic industry, the first MEMS devices were commercialized in early 1980, when MEMS pressure sensors and accelerometers were widely applied in the automotive industry [3,8]. In 1995, Bergveld first introduced the possibility of large-scale equipment miniaturization for chemical analysis [9]. As MEMS devices are currently present in all aspects of the daily life (e.g., image [10], touch [11,12], distance [13], pressure [14,15,16,17,18], temperature [19,20,21], and humidity [22,23,24,25] sensors, microphones [26,27,28,29], gyroscopes [30,31,32,33], accelerometers [34,35,36,37], and magnetometers [38,39,40]), the worldwide MEMS market is expected to grow to $25 billion by 2022 (Figure 1) [3,4,41,42,43].

The most common type of MEMS are transducers, either sensors or actuators, which convert one type of signal into another type of signal [3,44,45]. However, they can also be manufactured into cantilever or string forms, corresponding to single- or double-clamped beam-like structures, respectively [46]. The mechanism of the MEMS devices is based on energy transduction, and it involves the transfer of the information from the sensing unit to the controller, which will decide based on the control algorithm and further output the command to the actuating unit [45,47].

MEMS devices are highly advantageous, especially due to their small size, closely related to characteristics such as ease of integration, lightweight, low power consumption, and high resonance frequency [3,4,6,48,49]. Furthermore, MEMS offers the possibility of integration with electrical or electronic circuits, which increases their performance and makes them ideal candidates for self-powered and wearable electronics [3,50]. Other advantages include reduced fabrication costs due to high mass production and high accuracy, sensitivity, and throughput [4,6,48,49]. However, there are still some challenges regarding their manufacture, as MEMS devices are small, fragile, and sensitive and, thus, are subject to cracks, bending, or decalibration of the moving parts [51]. Additionally, due to complex mechanical geometries, MEMS devices are predisposed to failure due to particle contamination, fatigue, fractures, stiction of rubbing or contacting surfaces, and wear [52].

Traditionally, MEMS were used for the production of sensors, switches, filters, and gears [5,8] integrated with microelectronic, radiofrequency, optoelectronic, thermal, or mechanical devices [53]. Currently, they are applied in a wide variety of fields, including aerospace, automotive, military [4], microfluidics, energy harvesting and storage, data storage [5,53], telecommunications [6], analytical biology, and chemistry, and biomedicine [1,6,8,49]. In the medicine and health care systems, MEMS devices, also known as bio-microelectromechanical systems (BioMEMS), micro total analysis systems (µTAS) [54,55,56], lab-on-chips (LoCs) [57,58], or biochips [59,60], could be potentially applied in drug synthesis, drug delivery, microsurgery, microtherapy, diagnostics and prevention, artificial organs, genome synthesis and sequencing [44,48], and cell manipulation and characterization tools [44,48,61,62].

In this context, this paper aims to provide an overview of MEMS technology by describing the main materials and fabrication techniques for manufacturing purposes and their most common biomedical applications, which have evolved in the past years.

## 2. MEMS Fabrication Strategies

### 2.1. Materials

The fabrication of MEMS devices involves the use of semiconductor elements [2,63]. Similar to the integrated circuit field, silicon has been the predominant material for the manufacture of MEMS devices [9,64]. Specifically, doped single crystalline silicon and polycrystalline silicon, also known as polysilicon, are the representative materials in the field, owing to their unique properties, including strength, conductivity, high resilience, no stress hysteresis, robustness during fabrication, performance reliability, easy processing, and good process reproducibility, and low unit costs [65,66]. Additionally, silicon can also be used in the form of silicon dioxide for insulation and passivation or as a sacrificial material, and silicon nitride for insulation [2,65]. Although it is abundantly found in nature, silicon has gradually become insufficient for the continuously growing functionality and complexity of MEMS.

Therefore, new materials have been increasingly included in the fabrication of MEMS, namely single-crystal cubic silicon carbide, germanium-based materials, such as polycrystalline germanium and polycrystalline silicon germanium, metals and metal nanocomposites (e.g., titanium thin films, gold, aluminum, nickel-iron, titanium-nickel), polymer materials (e.g., polyimide, SU-8, parylene, polydimethylsiloxane, cyclic olefin polymers, polymethylmethacrylate or plexiglass, polycarbonate, polystyrene, liquid crystal polymers), ceramics (e.g., lead zirconate titanate, barium strontium titanate, aluminum nitride, gallium nitride), and piezoelectric materials [2,65,67,68,69,70,71]. For the adhesive component, there are three common materials involved, specifically 2025D, 3140RTV, and SDA6501. 3140RTV and SDA6501 are soft adhesive materials that are preferred over the 2025D hard material due to better mechanical isolation against thermal deformation [66,72].

Polymers have been growingly replacing silicon, as they possess advantageous complementary properties, including flexibility, easy processing, insulation, chemical and biological functionalities, biocompatibility, and low cost. Moreover, mixing polymers with filler materials can form composites with desired electrical, mechanical, and magnetic properties [66,73,74]. The most common applications of polymers in MEMS include structural materials, substrates, adhesives, sacrificial layers, and functional coatings [51]. Among the previously mentioned polymers, SU-8, polyimide, and parylene are more compatible with the conventional microfabrication techniques and have been widely used as free-film substrates and structural elements on hybrid silicon polymer devices [68].

Recent years have also witnessed an increase in the use of glass, particularly borosilicate glass, as a material for MEMS fabrication. It possesses exceptional properties that are well suited for this technology, especially in terms of proper hardness for suppressing channel wall deformation, superior optical transparency, chemical, and biological inertness, surpassing optical transparency and insulating properties, high solvent compatibility, and the possibility of surface modification for facilitating liquid flow [75,76]. However, the manufacturing of glass microfluidic devices is significantly expensive and time-consuming, involving complex, multi-step processes that combine different techniques, tools, and equipment [77].

Piezoelectric materials are classified into inorganic materials, which include lead zirconate titanate, aluminum nitride, zinc oxide, barium titanate, lithium niobate, and quartz, organic materials, including polar polymers, such as polyvinylidene fluoride, and optically active polymers, such as poly-L-lactic acid and poly-D-lactic acid. Although piezoelectric polymers are more cost-efficient regarding material cost and processing, inorganic piezoelectric materials have an enhanced piezoelectric output [63].

### 2.2. Microfabrication Techniques

Generally, electronics are fabricated through integrated circuit processing sequences, including metal-oxide-semiconductor processes or bipolar metal-oxide-semiconductor processes [78]. However, for the fabrication of MEMS devices, advanced technologies, processes, and materials are being used, as they require high-yield, affordable, and low-cost techniques to allow the development of 3D microscale structures [79]. The main technology for developing MEMS is semiconductor micromachining, which involves patterning through photolithography and etching for obtaining the required shapes [48,80,81]. Generally, patterning is the essential step in MEMS fabrication, which is performed through photolithography processes. Specifically, after exposing the light-sensitive polymer film-coated substrate to UV light, the polymer will change its solubility by becoming either more cross-linked if they are negative photoresists or less cross-linked if they are positive photoresists. The exposed areas are further developed away, resulting in a patterned film on the substrate [80,81,82,83].

**Figure 2:**
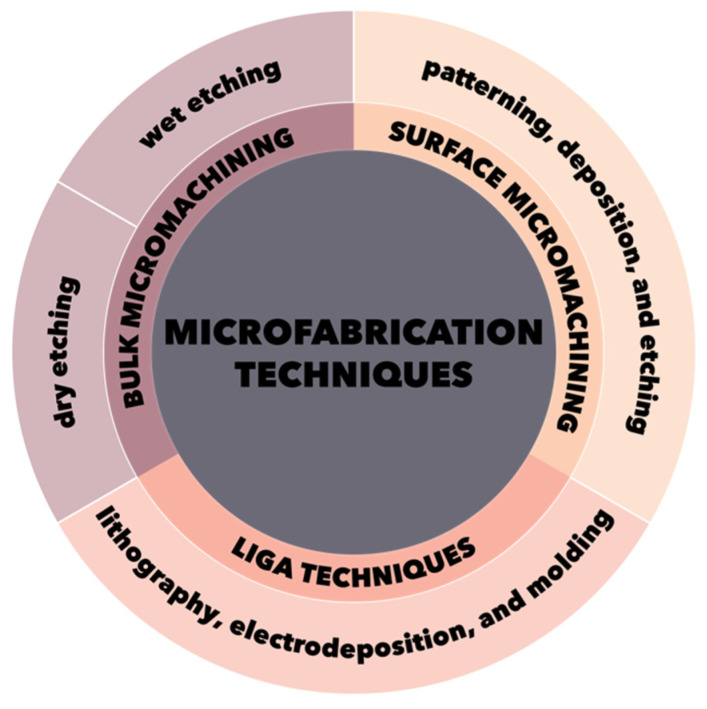
Schematic representation of the three main microfabrication techniques categories.

The main techniques involved in the construction of MEMS devices are lithographic and non-lithographic technologies [79,80,84,85,86]. The most common techniques are lithographic, which further involve three major categories, namely bulk micromachining, surface micromachining, and LIGA techniques (Figure 2) [79,80,84,85,86].

#### 2.2.1. Bulk Micromachining

**Figure 3:**
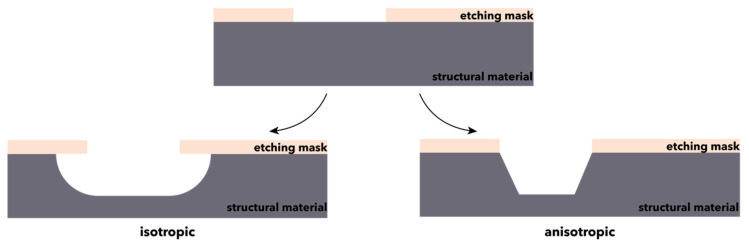
Schematic representation of the bulk micromachining processes.

Bulk micromachining is a process that involves the shaping of bulk materials through the treatment with orientation-dependent or -independent etchants [86]. It is a high-volume production process, where the back of a silicon wafer with a thickness in the range of 250–500 µm is isotropically or anisotropically etched to obtain the prescribed mechanical structure on top (Figure 3) [84,85]. To overcome the inherent limitations of the process, bonding methods are used for joining thin, 2D components into complex, thicker, and 3D structures [86,87]. Bulk micromachining is widely used for the development of undercut structures, such as membranes, grooves, beams, and holes for obtaining microstructures, i.e., accelerometers, pressure sensors, and flow sensors [81,84,85,86]. Based on the phase of the reactants, there are two bulk micromachining techniques that can be distinguished, namely wet and dry etching [81,86].

Wet etching, also termed chemical etching, is a simple and low-cost technique, as it is based on the reaction of liquid chemicals, mostly acids, and bases, with unwanted materials, which are removed from the top of multilayers when the materials are characterized by etching selectivity. Specifically, after the resist is applied and patterned over the material, a liquid chemical is used to etch the material selectively, thus remaining only the areas covered by the resist [88,89,90]. The generally used wet etchants include nitric acid or hydrofluoric acid, which will lead to the isotropic etching of the material, and potassium, sodium, or tetramethylammonium hydroxide for the anisotropic etching of the material [91,92]. Subsequently, using an appropriate solvent, the resist is stripped of the material. Although it is widely implemented in many manufacturing industries, wet etching is limited by the lack of control regarding fine-line patterning with critical dimensions [88,89,90]. A relatively more advanced wet etching technique is the Etching Microwave Silicon (EMSi), which employs microwaves for a faster process due to the heating of the potassium hydroxide etchant [93].

Dry etching, also termed plasma etching, is a technique performed in a gaseous phase that does not use liquids. Although it is sometimes referred to as reactive ion etching, the correct form is ion-assisted chemical vapor etching [89]. The dry etching process employs several steps, namely the generation and transport of reactive species (e.g., atoms, molecules, or ions) using ion bombardment or laser ablation within the vacuum chamber; the physisorption or chemisorption of the reactive species onto the surface of the material; the dissociation of the reactants and the subsequent formation of chemical bonds with the surface; the desorption and transport of the product species from the surface to the plasma [88,94]. Additionally, the plasma photons can also be adsorbed onto the surface, thus favoring the reaction and the desorption process by providing energy [95]. Since the ions are bombarded at a normal incidence to the surface, the etch profile is strongly anisotropic [89]. However, the isotropic etching of the material can be ensured through the use of xenon difluoride [91]. Dry etching allows for more precise control of the critical dimensions, thus being one of the most common techniques used in the integrated circuit industry [88,96]. However, there are still some challenges to consider when applying dry etching, such as the possibility of damaging the material [88] and profile distortion and mask sputtering due to excess physical or chemical processes [95,97]. As plasma processes are responsible for the characteristics of the MEMS devices in terms of performance, packing density, or power consumption, they must be further optimized to improve the final outcome of the applications [98].

#### 2.2.2. Surface Micromachining

**Figure 4:**
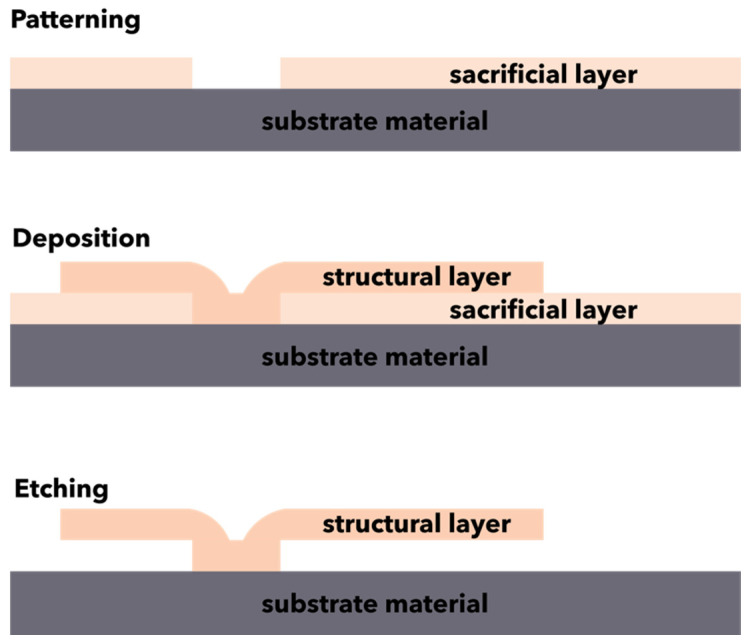
Schematic representation of the surface micromachining processes.

Surface micromachining is a technique that allows for the fabrication of microstructures through the alternative deposition, patterning, and etching of structural and sacrificial thin layers (Figure 4) [81,84,85,99]. The mechanism of surface micromachining typically involves the growing and patterning of a sacrificial layer onto a substrate, followed by the deposition of a structural material through physical or chemical deposition techniques (e.g., chemical vapor deposition, thermal oxidation) to generate films with thicknesses in the range of 2–20 µm. Subsequently, the structural materials are shaped through lithographic or etching techniques, and the sacrificial layer is removed using an appropriate wet etchant to form undercut, free-standing, movable microstructures [84,85,86,99]. Additionally, an intermediary step involving a high-temperature treatment called annealing could be performed to reduce the internal stress [99].

The choice of materials for developing these layers is generally based on the etching selectivity, as the sacrificial layer must easily be removed without affecting the structural layer. The structural layer usually consists of polysilicon, while the sacrificial layer can be made of silicon oxide, polymethylmethacrylate, or aluminum. Common etchants are acidic, such as hydrofluoric, phosphoric, nitric, or acetic acids [86,91,99].

Compared to bulk micromachining, surface micromachining is a highly advantageous technique, as it allows for the fabrication of structures as small as 10 mm, with smaller footprints and tighter tolerance, which are easier to integrate into electronic microstructures [86]. Therefore, it is a well-established technique for the mass production of MEMS devices, such as sensors, actuators, motors, gears, grippers, and micromirror assays [84,85,100]. However, surface microstructures handling and integrating during manufacture is challenging as they are considerably vulnerable [86].

#### 2.2.3. LIGA Techniques

LIGA represents the acronym for the German terms Lithographie, Galyanik, Abformung, which translate to lithography, electrodeposition, and molding [84,85,101,102,103]. Developed at Karlsruhe nuclear research center in Karlsruhe, Germany, in 1990 [103], LIGA technology is widely used for the fabrication of microstructures through the consistent application of deep X-ray lithography [101,102].

**Figure 5:**
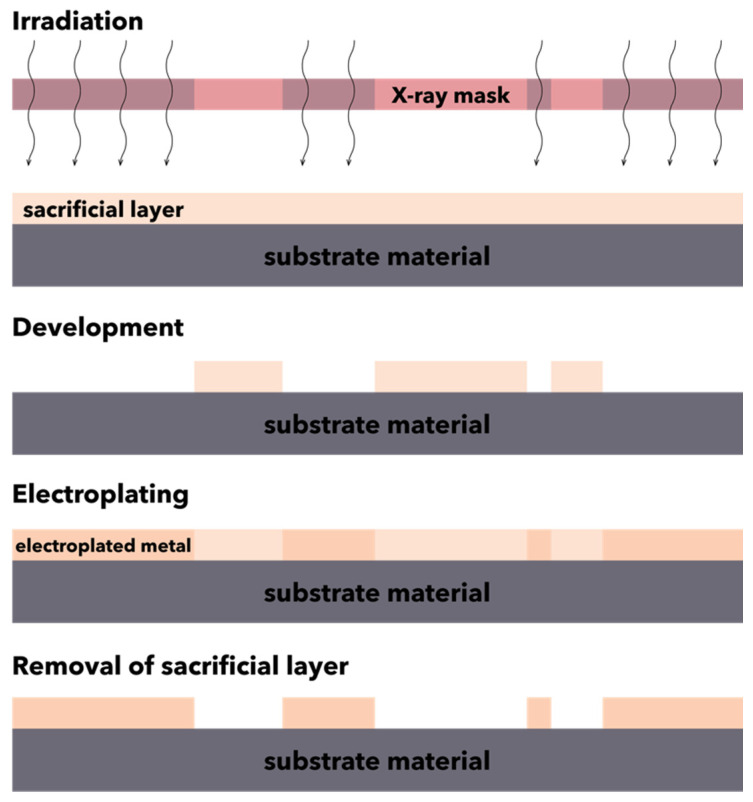
Schematic representation of the LIGA process.

The LIGA technique involves a series of steps, starting with the preparation of the X-ray mask using a thin film of gold to prevent the transmission of X-rays to the photoresist material outside the patterned area and the preparation of the substrate by using collimated deep X-ray lithography to illuminate and develop the desired pattern profile onto the thick film of photoresist material, such as polymethylmethacrylate, acrylic glass, plexiglass, polycarbonate, polyvinylidene fluoride, polysulfone, polyether ketones, or epoxy phenol resin. The photoresist material is placed on an electrically conductive or electrically conductive-coated insulating base material, i.e., austenitic steel, titanium, nickel, or silicon wafers coated with thin titanium, copper, or gold films, to facilitate the electrodeposition over the photoresist mold. Subsequently, the substrate material placed beneath the X-ray mask is exposed to the short wavelength X-ray radiations, resulting in a pattern profile with sharp, thin, and deep cavities of the photoresist. Further, the mold is formed through the melting of the photoresist, and the work material is electrodeposited into the mold. Finally, the excess electrodeposited material is removed, and the surrounding photoresist is removed or stripped from the final product (Figure 5) [103].

To optimize the processes involved in LIGA techniques, the properties of the materials must be completely known, as they are strongly correlated to the parameters of the process. Furthermore, the properties of materials and surfaces must be tailored with respect to the mechanical and optical properties, thermal and electrochemical stability, and biocompatibility of the resulting devices [104]. Specifically, the substrate material employed for the LIGA process should be characterized by high resistance to dry, and wet etching, thermal stability, adhesion tendency to the substrate during electrodeposition, and insolubility of the unexposed resist during fabrication [103].

LIGA technology represents a well-established technique for the mass production of microstructures implemented in micromechanics, microchannel systems, micro-optics, and X-ray micro-lenses. It offers a series of advantages over the other microstructuring technologies [102]. Specifically, it is possible to develop high precision and high aspect ratio (i.e., ratio between the height and the minimum lateral dimension) microstructures with flexible geometries and nanometer scale surface finishes (with a <50 nm surface roughness average value). Additionally, it allows for the use of a broad range of materials without introducing any thermal distortion and advanced scanner systems [81,102,104,105,106]. However, several limitations must be considered, namely the radiation threat to the operator due to the use of high energy X-rays, the difficulties in managing the multistep process, and the high costs of the equipment [105].

#### 2.2.4. MEMS Bonding Methods

**Figure 6:**
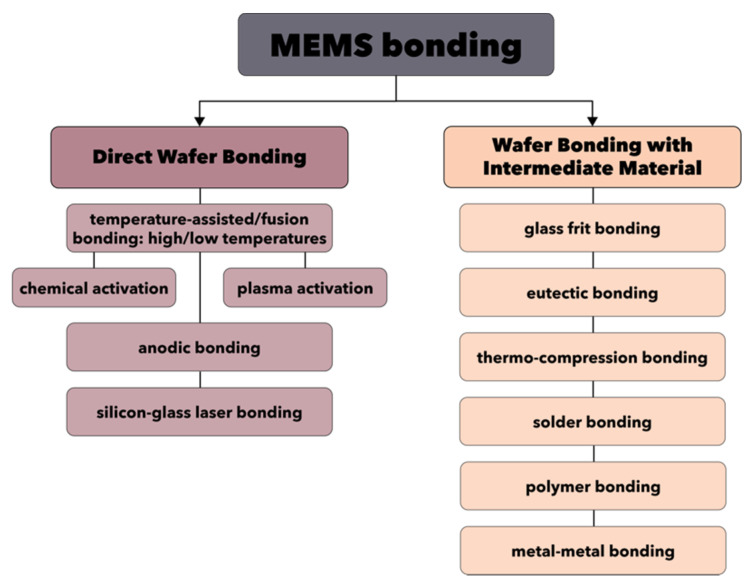
The main types of MEMS bonding techniques.

Another fundamental step involved in the fabrication of MEMS devices is the wafer bonding, which enables the possibility of obtaining complex and sophisticated 3D structures through the sealing or encapsulation of MEMS components. There are two main strategies involved in wafer bonding, namely direct wafer bonding and wafer bonding with intermediate material (Figure 6) [107].

On one hand, direct wafer bonding represents the most commonly applied technique, but also one of the most difficult to implement. Its success is essentially dependent upon the surface conditions and properties of the material, especially in terms of cleanliness (i.e., particle-free environment, since 1 μm diameter particles could generate 0.5 mm voids), roughness, and curvature. Additionally, as it might involve heat treatment steps, interfacial bubbles are often produced [107,108]. Generally, the direct wafer bonding process involves four main steps, namely bonding surface preparation, room temperature bonding, heat treatment, and wafer thinning [108]. As it can be seen in Figure 6, there are three types of direct wafer bonding techniques. First, the temperature-assisted direct wafer bonding, also known as fusion bonding, can be performed at both high or low temperatures and involves surface activation through chemical or plasma processes. Second, the anodic bonding is an electrochemically assisted technique performed at lower temperatures, which can rarely include the sputtering of an intermediary material, e.g., Pyrex. Third, the silicon-glass laser bonding is a technique that relies on laser-assisted direct wafer bonding.

On the other hand, the wafer bonding through intermediate materials is based on the deposition of glass, metallic, or polymeric intermediate films, followed by the actual bonding of the materials. The most common techniques are glass frit bonding and eutectic bonding, but they also include thermo-compression bonding, solder bonding, polymer bonding, and metal–metal bonding [107,108,109].

## 3. MEMS-Based Microfluidic Devices

Microfluidics is the science of fluid manipulation in systems with dimensions in the micrometer and nanometer scale. It has witnessed tremendous advancements over the last years owing to its broad application in various fields, such as biology, medicine, chemistry, and physical sciences [110]. Such systems have outperformed their ancestors by allowing for novel functionalities and studying phenomena elusive to the macroscale systems [111]. Although academically, it is a subdiscipline of fluid mechanics, the length scales in microfluidics are significantly reduced, and the fluid physics of such systems is considerably different. While the prerequisites to consider a system as being microfluidic are not clearly defined, it is generally accepted that one of the characteristic length scales, i.e., height or width, must be in the micrometer range or below [112].

**Figure 7:**
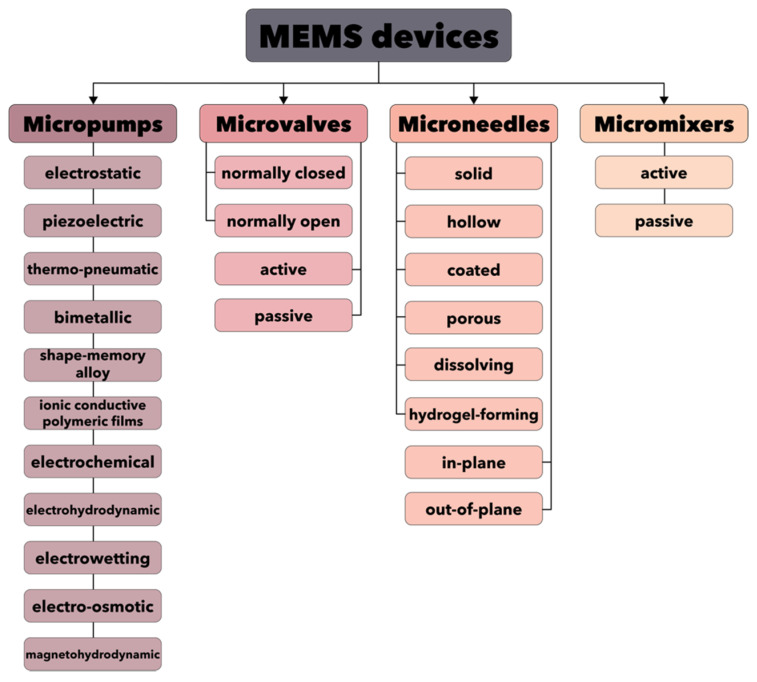
Classification of the main components for MEMS devices.

The recent progress in computational techniques has enabled the implementation of microfluidics for the design of more advanced MEMS devices. This technology is of fundamental importance as it poses a series of advantages, such as low energy and reagent consumption and high detection sensitivity [113,114]. Specifically, novel concepts have emerged, including LoCs, µTAS, or BioMEMS, allowing for the integration of entire biology or chemistry laboratories on a single chip [110,115,116]. This level of miniaturization has contributed to bridging the gap between the macro and microscale, which is the key element for the fabrication of automated, high-performance devices for screening and diagnosis, drug formulation and delivery, organ-on-a-chip, and microbioreactors [115,116,117,118]. Therefore, a wide range of microfluidic devices based on MEMS technologies has been developed, including, but not limited to, micropumps, microvalves, microneedles, and micromixers (Figure 7) [119,120]. Furthermore, the large number of manufacturing companies (e.g., Fluigent, Bruker, Schwarzer, Bartels Mikrotechnik GmbH, Darwin Microfluidics, Schott Minifab) focusing on producing these types of MEMS devices represents proof of the importance of this field.

### 3.1. Micropumps

**Figure 8:**
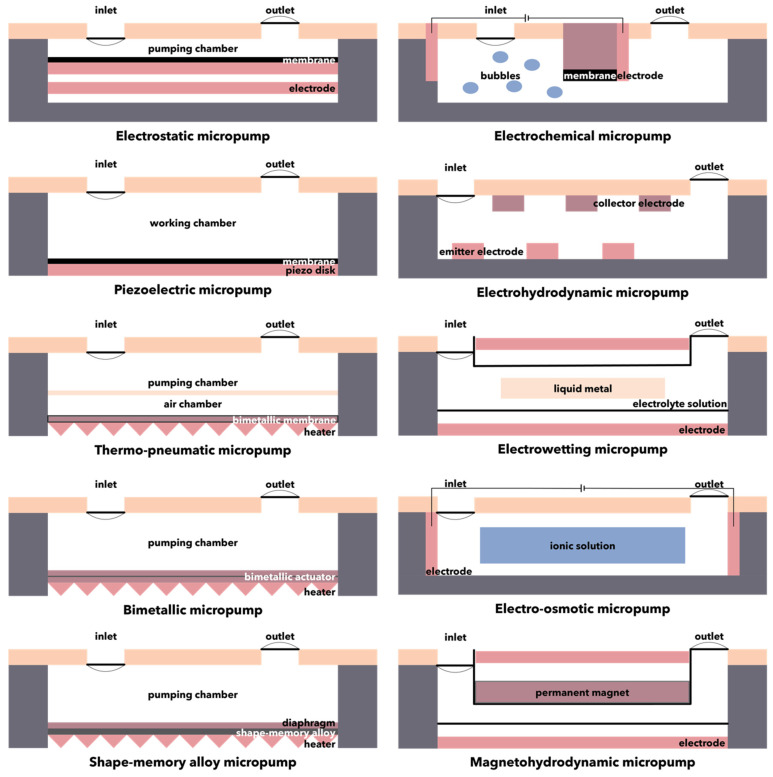
Schematic representation of the mechanisms involved in micropumps.

Applications such as advanced drug delivery, microchannel cooling, gas chromatography, miniaturized chemical analyses, and mobile-on-a-chip require precise fluid volume control at a certain flow rate. In this context, micropumps are microscale pumping devices that exhibit specific flow rates, pressure differences, and low power consumption, thus offering a solution for these applications [121,122]. Micropumps are usually manufactured through MEMS technologies, using biocompatible substrates composed of silicon, glass, polymethylmethacrylate, polydimethylsiloxane, or SU-8 photoresists [120]. Generally, they can be divided into mechanical micropumps, which contain mechanical moving parts such as valves or diaphragms for applying forces to working fluids through moving boundaries between the solid and liquid phases, and non-mechanical micropumps, which convert a considerable amount of non-mechanical energy into kinetic momentum to move the fluid along the channel [120,123,124]. Mechanical micropumps require different types of actuation mechanisms, such as electrostatics, piezoelectricity, thermo-pneumatics, bimetallic electro-thermal expansion, shape-memory effects, or ionic conductive polymer films (Figure 8, Table 1) [125,126,127]. As non-mechanical micropumps do not include any moving parts in their design, certain mechanisms for converting the energy into a kinetic momentum must be introduced. Hence, various engineering principles have been employed, including electrochemical, electrohydrodynamic, electrowetting, electro-osmotic, and magnetohydrodynamic mechanisms (Figure 8, Table 1) [121,126]. There are several key parameters that could affect the performance of non-mechanical micropumps. Specifically, the intensity of the electric field, which is dependent on the applied voltage, the frequency, and profile of the traveling wave, the type of fluid, the number of traveling wave phases, the gap size between the electrodes and their width, and the dimensions of the channels must be considered when designing non-mechanical micropumps [124].

#### 3.1.1. Electrostatic Micropumps

Electrostatic micropumps function with an out-of-plane mechanism based on the electrostatic forces that attract or repel the membrane clamped at its perimeter. Specifically, liquid pumping occurs when alteration pressures are created within the pump compartment due to membrane bending [122,126,128]. The scalability of the drive mechanism, facile manufacture, low power consumption, and fast response time are advantages of the electrostatic micropumps that make them attractive candidates for many applications. Nevertheless, in such systems, the design of the diaphragm and the drive is considerably challenging, as it is necessary to reach a sufficient stroke volume for the pumping effect and a failure-free operation, which are further complicated by the small deflections of the microactuators [122]. Additionally, drawbacks associated with the high actuation voltage and the pull-in instability must also be considered [129].

#### 3.1.2. Piezoelectric Micropumps

The motion of the fluid within piezoelectric micropumps is realized by the deformation of the pump chamber, which generates a pressure difference due to the motion of piezoelectric elements. Since most piezoelectric micropumps use a piezoelectric plate or disk glued directly on the pump diaphragm and work in a quasistatic state, the working frequency and the volume change induced by the deflection of the diaphragm are reduced [130]. These pumps have been broadly applied as they pose a series of advantages, such as small size, low power consumption, no electromagnetic interference, and insensitivity to the fluid viscosity. Among the piezoelectric materials commonly utilized for their fabrication, lead zirconate titanate ceramics have demonstrated optimal performances [129].

#### 3.1.3. Thermo-Pneumatic Micropumps

The thermo-pneumatic micropump is a mechanical micropump that functions through the intermittent compression and expansion of a compartment filled with air by a cyclic use of a twosome heater and cooler. This intermittent volume delivers a recurrent impetus for fluid flows, which creates a considerably large induced pressure and a membrane displacement [128,131]. To initiate the transfer of heat from the human body to the micropump, several mechanisms have been implemented, such as body heat-powered or fermentation-powered operations [132,133].

#### 3.1.4. Bimetallic Micropumps

The mechanism of the bimetallic micropump is similar to the thermo-pneumatic micropump, as it consists of two materials with different thermal expansion coefficients. The actuation of the micropump is provided by the repeated heating and cooling cycles that lead to thermal stresses [48,134]. The advantages of bimetallic micropumps include the generation of high forces, low operating voltage, and simplicity of the design. However, small-diaphragm deflection and unsuitability at high frequencies limit the applicability of such systems [134].

#### 3.1.5. Shape-Memory Alloy Micropumps

The shape-memory effect represents the recovering of a material’s original shape from its distorted shape after heating or cooling, and it includes a process of phase alteration inside two solid phases, namely the austenite high-temperature phase and the martensite low-temperature phase [128]. Shape-memory alloys have attracted significant research interest due to their shape memory effect and superelasticity. In the biomedical field, nickel-titanium shape-memory alloys are the ideal candidates owing to their biocompatibility, resistance to corrosion and fatigue, and elastic modulus that is similar to that of the human bone. Although pure nickel is known to be a toxic element for the human body, nickel–titanium alloys are considered safe. Additionally, nickel can be partially replaced by copper, cobalt, iron, niobium, and molybdenum to improve stress and/or temperature hysteresis, corrosion and fatigue behavior, and control of transformation temperatures. Shape-memory alloy micropump-based MEMS devices have been investigated for various biomedical applications, such as endovascular surgery, intestinal obstruction, neural interfaces, and drug delivery [135,136].

#### 3.1.6. Ionic Conductive Polymeric Films Micropumps

Ionic conductive polymeric films micropumps are generally composed of a polyelectrolyte film with both faces chemically plated with platinum and are actuated by the stress gradient of the ionic movement generated by an electric field. Specifically, the cations within the electrically conductive films will take water molecules and move to the cathode by applying an electric field. Consequently, the cathode will expand, and the anode will contract. However, if an alternating voltage is applied, the diaphragm attached to one end of the film will bend alternatively. Such systems are commonly called artificial muscles owing to their large bending displacement, low driving voltage, low power requirements, rapid reaction, and high biocompatibility. Thus, various applications utilizing ionic conductive polymeric films micropumps have been reported, including robotics, micromanipulators, and medical devices. Nonetheless, the fabrication process of these micropumps is highly complex, and the level of reproducibility during batch manufacturing is considerably low [128,134].

#### 3.1.7. Electrochemical Micropumps

The actuation mechanism of electrochemical micropumps is based on the reversible electrochemical reactions that allow for the expansion of gas bubbles and the reduction through electrolysis in the aqueous electrolyte solution. These systems are highly advantageous as they provide large driving forces, accurate flow control, low heat generation, compliance with LoC technologies, and low power consumption. However, their application is limited by the long response time caused by the slow recombination of the gas within the working chamber [137]. While previous devices allowed for the monitorization of the fluid delivery using wired, electrochemical micropumps, recent studies have demonstrated the applicability of wireless implantable pumps to eliminate the need for transcutaneous wires and catheters and thereby reducing the complexity of the surgery, to improve the mobility of the patient, and to allow for drug administration outside the healthcare facility [138].

#### 3.1.8. Electrohydrodynamic Micropumps

The electrohydrodynamic micropump is actuated through the interaction of electrostatic forces with ions within dielectric fluids. Therefore, fluid motion necessitates the presence of an electric field, obtained through the exertion of a potential difference to an array of electrodes to produce a traveling wave and a space charge, which is achieved by using non-homogeneous fluids or by the dissociation or direct charge injection. In this manner, the mechanisms are further classified into induction, conduction, and electrohydrodynamic injection pumping, respectively [124].

#### 3.1.9. Electrowetting Micropumps

Electrowetting micropumps are based on a microfluidic phenomenon through which the surface energy of a conductive liquid changes in contact with a dielectric-coated electrode when an external voltage potential is applied [126,139,140,141]. This process is reversible, as the system returns to the original configuration when the potential is removed [126]. This approach is recognized as one of the most flexible and effective tools which allow for the manipulation of discrete liquid volumes with increased reproducibility, mobility, and reversibility [126,139,141]. However, under low voltage input, electrochemical interactions easily occur due to the direct contact between the liquid and the electrode [141].

#### 3.1.10. Electro-Osmotic Micropumps

Electro-osmotic micropumps consist of a microchannel with electrodes submerged in fluid reservoirs at one end to achieve high pressure for driving liquids through the field-induced ion drag mechanism. Specifically, the working principle of these pumps involves an electrokinetic effect known as electro-osmosis or electro-osmotic flow, through which the uncharged liquid moves relative to the charged microchannel surfaces under the action of an externally applied electric field [126,142]. Electro-osmotic micropumps possess the advantages of non-mechanical micropumps and electrically controlled methods, as there are no moving parts, and they allow the use of fluids with a wide range of conductivities [142,143]. However, electro-osmotic micropumps are not suitable for electrolytic aqueous solutions, which are widely applied in microfluidics [143].

#### 3.1.11. Magnetohydrodynamic Micropumps

The working principle of magnetohydrodynamic micropumps involves the magnetohydrodynamic phenomenon through which an electrically or weakly electrically conductive liquid will be driven through the microchannels by the Ampère’s force resulting from the interaction between the charged conductor and the external magnetic field [143,144]. These pumps are widely advantageous and have been used in various microfluidic applications, including liquid chromatography, integrated fluidic networks, or mixing [143].

### 3.2. Microvalves

Furthermore, microvalves represent an essential component of MEMS-based microfluidic devices which can ensure sealing, on/off switching, and fluid flow regulation. In this context, various types of microvalves have been developed in order to fulfill different application-specific tasks [145,146].

**Figure 9:**
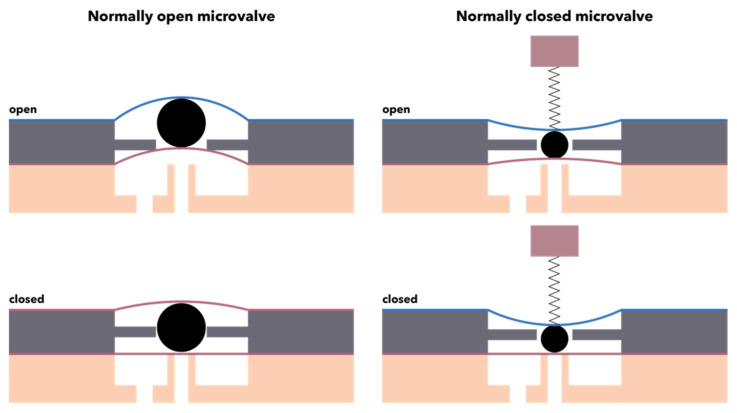
Schematic representation of normally open and normally closed microvalves.

On the one hand, microvalves can be categorized based on their initial mode, namely normally closed, requiring the simplest fabrication techniques, normally open or bistable, that is generally preferred due to their low power consumption (Figure 9). On the other hand, microvalves can be classified based on their working mechanism into passive or active. Passive microvalves do not require any energy source to control the flow within the microchannels, as their functioning depends on their geometrical configuration or the utilization of mechanical or non-mechanical moving parts, e.g., diffusers or flaps. While they represent a low-cost option due to low power consumption, passive microvalves are associated with a variety of limitations in terms of efficiency, performance, and leakage [145,146]. Therefore, research has been shifted towards implementing active components in a cost-efficient and portable manner [146]. By contrast, active microvalves utilize an external energy source or system for actuating the mechanical and non-mechanical moving parts, thus generating large forces and faster response times [145]. The working principle of active mechanical microvalves involves coupling a thin deflectable membrane to microactuators with different mechanisms, including pneumatic, piezoelectric, electrostatic, magnetic, or thermal, while non-mechanical microvalves generally use smart materials, such as rheological or phase change materials [145,146,147,148]. Owing to their facile integration that allows for the precise control of fluids, the pneumatic actuation-based microvalves are the most commonly used ones. Their functioning relies on the deflection of an elastomer material membrane, e.g., polydimethylsiloxane, that can interrupt the flow through the microchannel [147,148].

### 3.3. Microneedles

Microneedles consist of small microprojection arrays comprising micron-scaled needles attached to a support with lengths in the range of 25 to 2000 μm [149,150]. They were first introduced in 1976, with an American patent involving the application of microneedles for transdermal delivery released simultaneously [151].

In this context, microneedles might be considered a microscaled hybrid between transdermic patches and hypodermic syringes, as they are able to ensure transport pathways between the microneedle array and the biological membrane [149,150]. Therefore, with diameters considerably larger than molecular sizes, they can be applied to deliver submicronic molecules, macromolecules, supramolecular complexes, and particles. Specifically, microneedles could deliver small and large molecule drugs (e.g., antibiotics, insulin), hormones, vaccines, proteins, peptides, and nanoparticles, thus enhancing drug efficacy through direct epidermis transfer and, consequently, long-term treatment, immunobiological administration, and diagnostics [149,151,152]. The advantages of microneedles within the biomedical field include reduced dosage frequency, minimum side effects, self-administration possibility, non-invasiveness, or minimal invasiveness, and, subsequently, improved patient compliance, first-pass metabolism, and gastrointestinal incompatibility avoidance, plasma drug level maintenance, improved bioavailability, and the possibility to administrate drugs with short half-lives and narrow therapeutic indices. Nonetheless, several limitations must be overcome, including reduced dose accuracy, skin penetration and delivery variations due to thickness differences and skin condition within each patient, and microneedle breakage or blockage [153].

**Figure 10:**
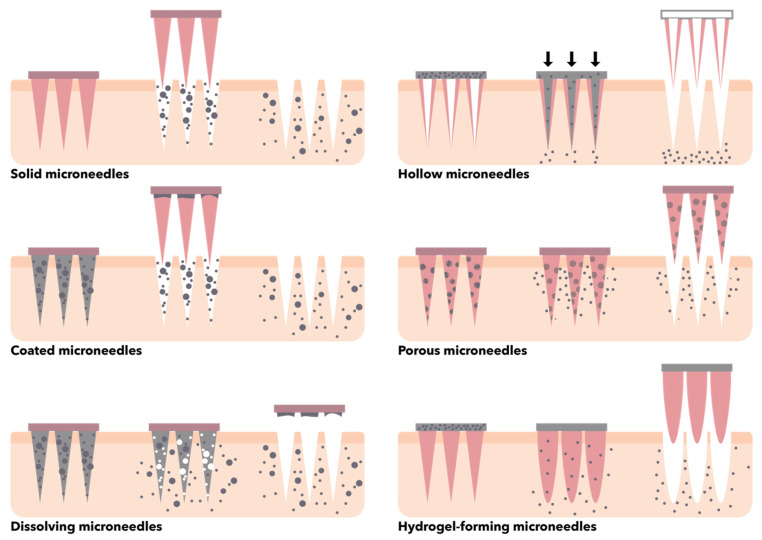
Schematic representation of the main types of microneedles based on their structure.

In this context, there is a wide variety of microneedle designs and configurations that have been investigated over time for different applications. Specifically, microneedles can be categorized based on their structure (i.e., solid, hollow, coated, porous, dissolving, hydrogel-forming) (Figure 10), outer structure (i.e., in-plane, where the length of the microneedle is parallel to the substrate plane, and out-of-plane, where the longitudinal axis of the microneedle is perpendicular to the plane), shape (e.g., canonical, cylindrical, pyramidal, pentagonal, hexagonal, octagonal, square, and candle-, spike-, spear-, rocket-, and star-like), and tip shape (e.g., canonical, cylindrical, tapered, volcano, and snake fang) [150,153,154,155]. Generally, the materials used for the fabrication of microneedles should be non-toxic, pharmacologically inert, compatible with the molecules to be delivered, non-corrosive, economical, easily available, user-friendly, and have high tensile and mechanical strength [150,153]. Thus, a variety of materials have been investigated for the fabrication of microneedles, including glass (e.g., silica glass, borosilicate glass), silicon, biodegradable and non-biodegradable polymers (e.g., polycarbonate, cyclic olefin copolymer, polyglycolic acid, polylactic acid, polylactic-co-glycolic acid, polymethylmethacrylate, polydimethylsiloxane, polyvinyl alcohol, polyvinyl pyrrolidone, silk fibroin, maltose, galactose, mannitol, sucrose, trehalose, xylitol), ceramics (e.g., alumina, zirconia, calcium sulfate dihydrate or gypsum, calcium phosphate dihydrate or brushite, Ormocer^®^), and metals (e.g., titanium, stainless steel, cobalt, nickel, palladium alloys) [101,150,153,156]. The microneedle fabrication methods are usually based on four main processes, namely patterning, deposition, etching, and bonding [157]. These can be performed by conventional microfabrication techniques for adding, removing, and copying microstructures through photolithography, laser cutting, micromolding, dry and wet etching, and metal electroplating and electropolishing [149,153,158].

### 3.4. Micromixers

Since they significantly influence the sensitivity and efficiency of microfluidic devices, micromixers represent one of the most important MEMS components [159]. Additionally, micromixers have proven their efficacy within the medical, biological, and chemical fields by ensuring rapid and accurate reagent detection and disease diagnostics and enhanced drug screening [160,161].

While macroscaled fluidic devices imply fluid mixing by convection effects, mixing within microfluidic devices is generally performed through external turbulences and/or specific microscaled structures that increase surface-to-volume ratios and efficiency of heat and mass transfer. Furthermore, as the fluid flow through MEMS devices is laminar, fluid mixing is mostly based on diffusion with reduced mixing efficiency [159]. The design of micromixers ought to consider optimal and specific parameters that will ensure the previously mentioned features, such as shortest length of the microchannel for complete fluid mixing, easy integration, and pressure drop, which improves fluid mixing but reduces device longevity and increases power consumption [161]. Micromixers can be categorized into active, which require external energy sources, such as electrical or magnetic, and passive, that are generally based on complex geometries of the microchannels for manipulating the laminar flow in chaotic advection-based micromixers or the increase in contact surface and time between the fluid layers for molecular diffusion-based micromixers. Since active micromixers require additional components and energy sources, their integration is more difficult and expensive. By contrast, passive micromixers are more stable, robust, and easy to fabricate and integrate within microfluidic devices [159,160,161,162].

## 4. Biomedical Applications of MEMS Devices

**Figure 11:**
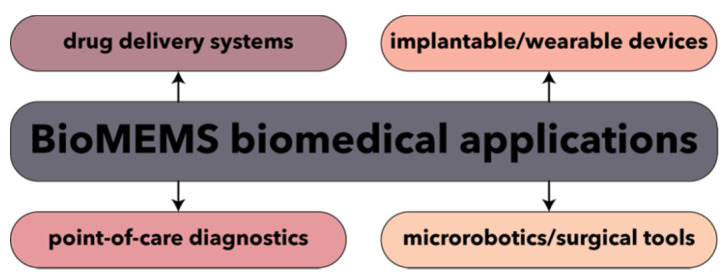
The main biomedical application fields for BioMEMS.

Recent years have witnessed enormous technological advancements in the field of biomedical MEMS, also known as BioMEMS, which have been promoted for the last 20 years as the ultimate microscaled tools for the ultrasensitive, precise, rapid, and low-cost alternative for disease diagnostics and management. Combining the knowledge of both MEMS and biology disciplines, BioMEMS have been designed and fabricated towards the development of point-of-care disease diagnostic devices and biosensors, drug delivery systems, and surgical tools (Figure 11) [163,164,165]. Moreover, MEMS technology has been widely used as platform for the synthesis of nanoparticles with enhanced and uniform characteristics [166].

### 4.1. Disease Diagnostics

The possibility to develop MEMS in the sub-micron range has allowed for their implementation in the form of user-friendly miniaturized diagnostic devices, also known as biosensors or biosensors-on-chip. Such devices represent promising solutions for overcoming the challenges of conventional diagnostics, such as time- and power consumption, possible contamination, and large sample size, while also ensuring multianalyte determination and higher sensitivity, accuracy, specificity, and precision of detection, identification, and quantification [163,164,167,168].

**Figure 12:**
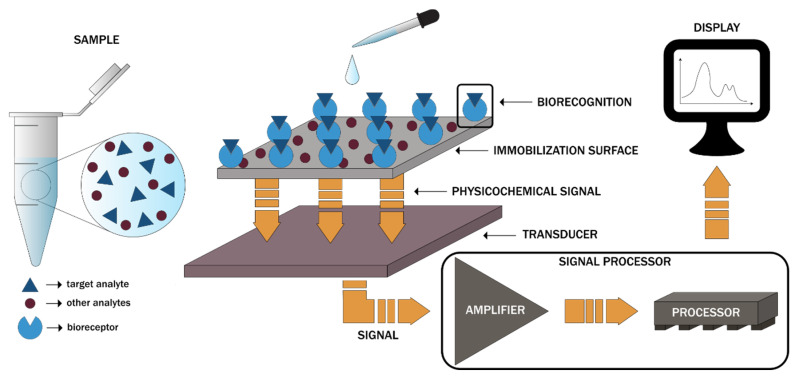
The working principle of biosensors-on-chip and the associated components [169].

Biosensors-on-chip represents micro-total analysis systems incorporating all the steps involved in sample analysis, namely injection, mixing, reaction, separation, enrichment, and detection, within a single device. While their design may contain a wide variety of units, the setup generally involves four main units, namely the inlet segment for sample injection, the reacting segment for the required reactions, the analysis segment for the detection of the reactions by the biosensors, and the data processing segment for the conversion of the resulting signals into the output signals (Figure 12). Each of the four segments contains specific MEMS components required for controlling and operating the fluids and chemical processes, such as micropumps, microvalves, micromixers, and microchannels [169]. Generally, body fluids, including blood, serum, urine, saliva, or liquid biopsies, are introduced within the microchip for disease diagnosis. In this regard, the literature reports involve the use of biosensors to detect and monitor glucose, hemoglobin, urea, amino acids, body gases, viruses, bacteria, and cancer biomarkers [164,167,169]. However, BioMEMS can also be used as devices for the constant and continuous measuring of blood oxygen, intraocular or aortic pressure, electrical impulses, or metabolic processes and can be either wearable or implantable [170].

**Figure 13:**
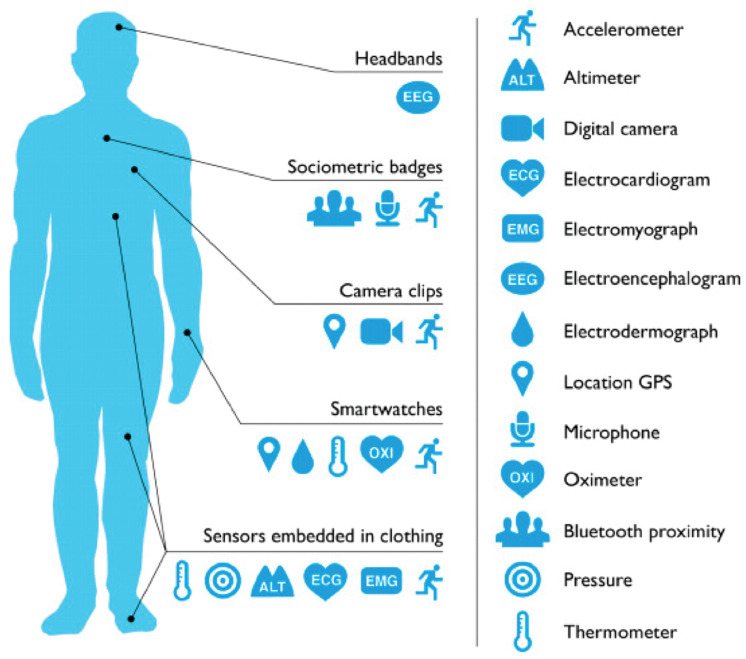
Schematic representation of wearable devices and the associated location on the human body. Reprinted from an open-access source [173].

Wearable MEMS have become a major part of the daily lives of chronic patients, as they are able to remotely monitor vital signs, such as blood and intracranial pressure, glucose levels, heart and respiration rate, body temperature, or O_2_ saturation [171,172]. In this context, a new notion of “quantified self” has arisen, referring to the process of personalized data collection regarding the patient’s wellbeing through wearable technology. Examples of such devices include headbands, wristbands, smartwatches, textile sensors, sociometric badges, and cameras incorporating MEMS devices (Figure 13), which could be able to manage stress, improve sleep patterns, increase productivity, or monitor the progression of the disease [173].

Recently, BioMEMS have attracted great interest for their use in diagnosing the novel coronavirus. As conventional techniques, namely the polymerase chain reaction (PCR) technique, generally imply several hours for reaching an outcome and can lead to numerous false-negative results due to the limited amount of sample collected, the emerging BioMEMS-based techniques could provide the fundamental support in the fight against the current and future pandemics [174]. Additionally, these techniques could include specific components responsible for the three main steps of PCR, i.e., hydrogen bond breaking through heating, reaction with the primer through culling down, and DNA replication with nucleotides and DNA polymerase through the second heating step, onto a single chip. In this manner, a cost-effective and rapid diagnosis device could be developed [175,176].

### 4.2. Drug Delivery

The precise and accurate delivery of drug amounts represents a key element of effective therapeutics. For every drug, the minimum effective concentration, which is the minimum amount of drug that must be present in blood plasma to elicit its therapeutic effect, and the maximum therapeutic concentration, defined as the plasma concentration above which the drug will cause toxic effects, represent two crucial parameters. As they are different for each drug and from patient to patient, the formulation of drug delivery systems with controlled release represents a serious challenge [163]. Thus, the last decade has witnessed an increase in BioMEMS-based drug delivery-associated research. Implantable or wearable systems with on-demand release have demonstrated promising results in the accurate and controlled release of drugs, which most conventional technologies fail to accomplish [163,177]. Implantable BioMEMS devices and MEMS technologies have provided the possibility of drug delivery in chronic and long-term diseases. Specifically, they have allowed for the customized dosage of insulin according to blood glucose levels in diabetes and the release of specific drugs in clinical conditions where sudden death might appear, such as myocardial infarction or septicemia [163,178]. Additionally, implantable BioMEMS could overcome the limitations of gene and peptide delivery, such as 3D structure maintenance for optimum activity, allowing for the delivery of nucleic acids and peptides at the target site [2].

**Figure 14:**
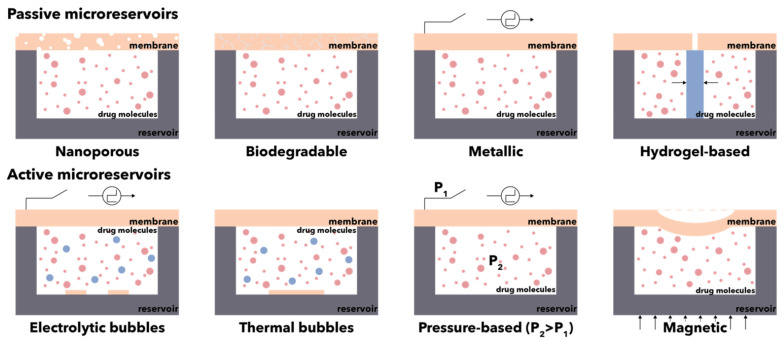
Schematic representation of the main types of microreservoirs for the controlled release of drugs.

Unlike conventional systems, which majorly rely on diffusion, implantable BioMEMS for drug delivery involves single or multiple drug-containing reservoirs that continuously control the release rates and infusion volumes (Figure 14) [179,180]. In this manner, the conventional drug administration routes which affect patient compliance, such as intravenous or intramuscular injections, are avoided [177]. Microreservoirs represent drug containers that ensure drug storage prior to its release, either slowly overtime through porous membranes or at specific time intervals by diffusion or expulsion [181]. Typically, such systems consist of five main components, i.e., micropumps, microreservoirs, micro-sensors, microchannels, and the control system [163]. The materials used for reservoir fabrication must be biocompatible on the outside and inert on the inside, as they come in direct contact with the administered liquid-phase drugs. Thus, owing to their biocompatibility, bonding, and optical transparency, polydimethylsiloxane, polyacrylamide, medical-grade silicone, and Pyrex© are the most widespread materials for the construction of reservoirs. Furthermore, the microfabrication process must allow for their fabrication with a size large enough for loading the suitable amount of drug but small enough to ensure the efficiency of the actuating mechanisms that will release the drugs. Additionally, considering their implantable destination, the total size of the device must not be considerably increased [179]. In this manner, the dosage-, temporal-, and spatial-controlled release of drugs can be ensured, without depending on the nature of drugs, the target areas, or the administration routes [180].

**Figure 15:**
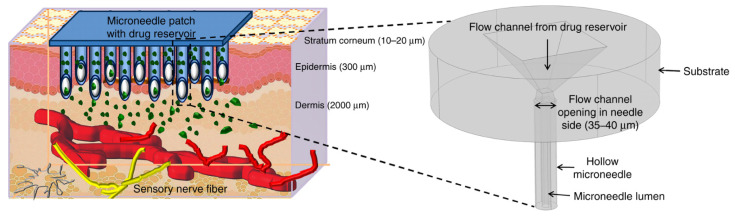
Schematic representation of the transdermal delivery of drugs through the use of microneedles.

Moreover, the external stimulus-triggered smart release of drugs has been intensively studied, which allows for the intermittent, as needed, and over prolonged periods dosage through wireless transmission [177,180]. Such advancements could enable patient-specific, pinpoint treatment strategies of various diseases, including cancer, osteoporosis, and diabetes, with reduced invasiveness due to significant miniaturization processes [182]. Moreover, BioMEMS, through the use of microneedles, could also ensure a highly effective transdermal delivery of drugs (Figure 15) [101,149,156,158]. This is a highly important feature, as they could overcome the issue associated with poor drug absorption or the enzymatic activity in the gastrointestinal tract or the liver that results in the degradation of the drug molecules [179].

Besides the release of drugs, implantable therapeutic BioMEMS are also able to provide programmed stimulations (e.g., based on abnormal electrocardiogram detection, electrical simulation BioMEMS, such as the implantable defibrillator, will generate real-time, programmed electrical signals) [178].

### 4.3. Microrobotics and Surgical Tools

**Figure 16:**
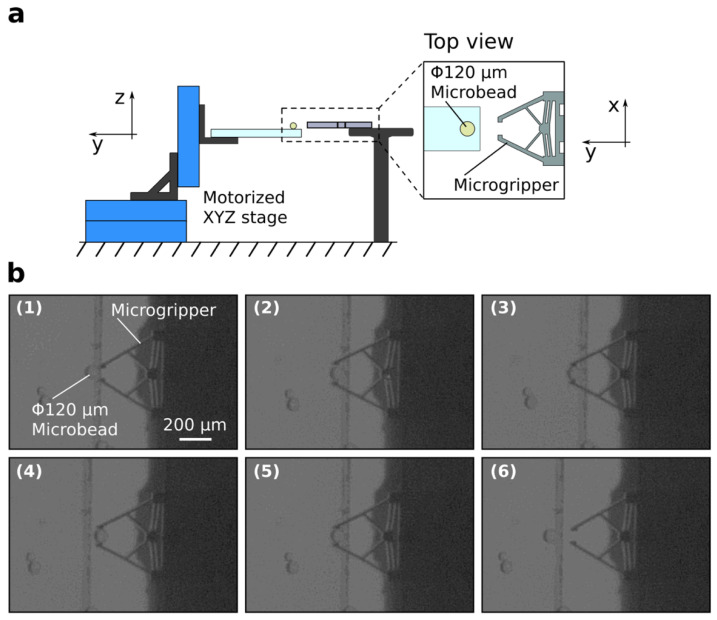
Schematic representation of the microgripper and the experimental setup (**a**). Images showing the successful pick-and-place action of a microbead ((**b**),(**1**)–(**6**)). Reprinted from an open-access source [188].

Surgical interventions represent the treatment of diseases and affections through the use of manual or instrumental means [167]. While they have considerably advanced throughout the past decades and are fundamental to the healthcare system, their invasiveness and potential scarring and trauma have determined scientists to constantly seek alternative possibilities or ways to minimize invasiveness [167,183]. The evolution of surgical procedures started from the first generation of open surgeries, which involved large incisions made by surgeons in the patients to access the site completely. While the surgeon directly viewed and felt the tissues and internal organs, such procedures resulted in considerable trauma, risks associated with anesthesia, and longer recovery periods [167,184]. Therefore, the second generation of minimally invasive surgery, also known colloquially as keyhole surgery, was born as a technique to access the desired sites through very small incisions using an endoscope. However, as they involve a camera that provides 2D images of the site, there is a loss of tactile feedback and depth, which overshadow the advantages of shorter hospital stay and reduced post-operative pain and discomfort [167,185]. In this manner, the birth of robotic surgery or robotic-assisted surgery as the third generation of surgery sought to eliminate such disadvantages. Specifically, this type of surgery manipulates joystick actuators on the computer console with a stereo display. It contains tremor cancellation algorithms to minimize any possible human error. The robot comprises three arms, one for the endoscope and two for maneuvering the surgical instruments [167,186]. It has multiple advantages, including small incision, reduced pain, blood loss, and infection risk, shorter healing periods, and high comfort of the surgeon, which subsequently prevents loss of attention [186]. However, scientists and engineers strive to improve and innovate robotic surgery to enhance vision, accessibility, and haptics [187]. In this regard, owing to their considerably reduced size and high performance, MEMS-based surgery tools have received great scientific interest. Through their application, issues related to tissue sensing, tactile feedback, instrument tracking, and maneuvering could be solved [167]. Specifically, surgical tools such as endoscopes, manipulators, imaging devices, and catheters could be developed using MEMS technologies, thus paving the way towards microsurgery purposes [182]. Figure 16 depicts the schematic representation of a microgripper and the images of the pick-and-place action of the microgripper [188].

### 4.4. Cell and Gene Manipulation and Characterization

**Figure 17:**
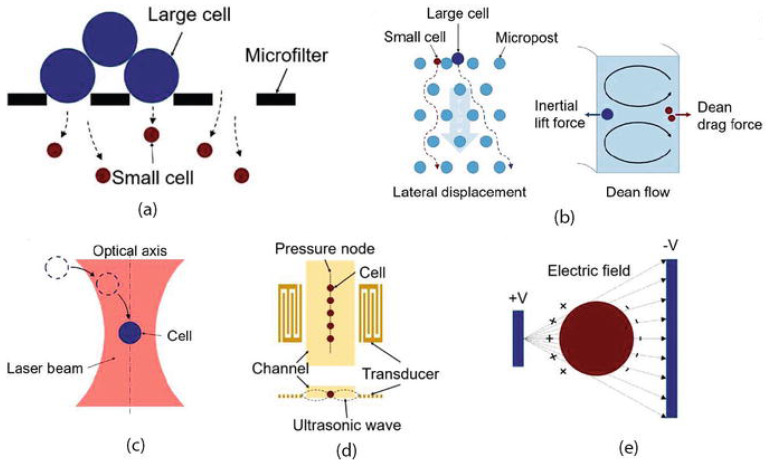
Cell separation mechanisms—geometry-based separation (**a**), affinity-based separation (**b**), optic separation (**c**), acoustic separation (**d**), electrokinetic separation (**e**). Reprinted from an open-access source [192].

**Figure 18:**
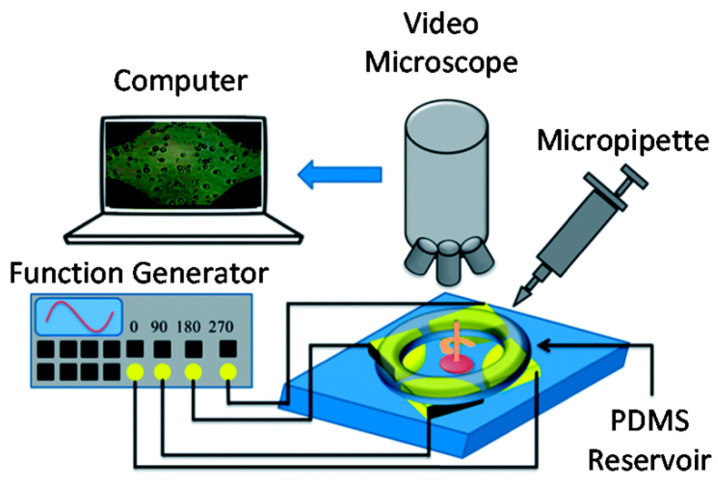
Schematic representation of the experimental setup for electrorotation tests using a BioMEMS components. Reprinted from an open-access source [189].

BioMEMS have also been adapted for the precise processing, manipulation, constructing, and analysis of biological entities [189]. Particularly, owing to their size matching of single cells, they have become the ideal tools for cell manipulation and characterization. Moreover, as MEMS possess the ability to generate and measure motions and forces within the microscale, they have been regarded as one of the most straightforward end-effectors and sensors. Their high resolutions below the nanometer and nanonewton levels, respectively, allow for the accurate detection of the smallest cell deformations and lowest cell forces (Figure 17) [190]. The manipulation of cells generally implies the application of differential forces onto the targeted cells in order to direct them along the desired path. Based on the mechanism of force application, the separation of cells can be classified into the geometry-based, affinity-based, acoustic, optic, and electrokinetic (e.g., dielectrophoresis (Figure 18) [189]) methods [191]. In this context, there are several examples of BioMEMS platforms for cell manipulation and characterization, such as cell grasping, transfer, and mechanical characterization microgrippers, cell force and mass/density measurement physical sensors, cell immobilization and patterning devices, and cell injectors [190].

**Figure 19:**
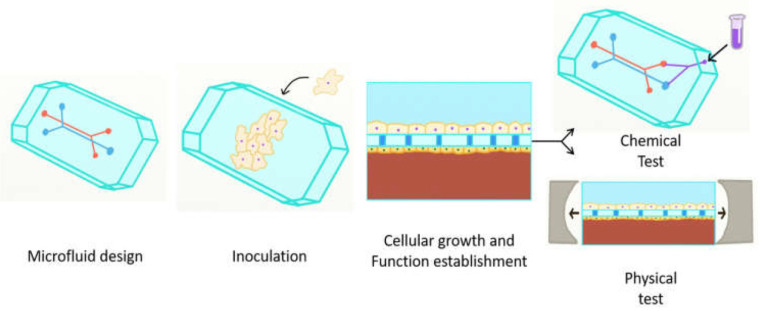
Schematic representation of the main principles involved in the design of organs-on-chip. Reprinted from an open-access source [196].

Furthermore, BioMEMS have also revolutionized the 3D cell culture technology, as the microenvironment ensured by these devices is highly compatible with the in vivo conditions. Specifically, the size and traffic matching of cells inside the organism, the chemical gradient mimicking the complex organ and tissue systems, the cost efficiency due to low reagent volume use, the biocompatibility of the substrates due to oxygen permeation that allows for cell growth and proliferation, and the possibility of concomitantly handling several processes (e.g., medium replenishment, detachment, sampling, mixing, capturing, detection) are the fundamental features that make BioMEMS unique for 3D cell cultures. Moreover, the BioMEMS-based 3D cell culture can be further applied for creating model human tissue/organ/body-on-chip systems (Figure 19). Such systems represent promising alternatives for the rapid testing of novel diagnostic and therapeutic molecules without involving any risk to animal models and human subjects [193,194]. While there are many literature studies investigating the development of organoids using microfluidic BioMEMS, the field is still in need of intensive research due to the complexity of the systems [195].

BioMEMS have also proven efficient in the field of gene analysis, namely DNA sequencing, gene expression analysis, and single nucleotide polymorphism genotyping. Additionally, they have been recently used for the development of aptamers which is of great importance for cancer therapy [191].

## 5. Challenges and Future Directions

The highly steep progression of MEMS technology in the 1980s and 1990s enabled by semiconductor integrated circuit manufacturing-derived microfabrication techniques is currently facing a series of challenges. Specifically, the currently available technologies will soon be insufficient for future requirements regarding the performance, functionality, size, power consumption, and costs [197].

One of the MEMS-associated challenges is the reliance on silicon and its derivatives as the sole types of materials [198]. In this context, novel advances in material science and engineering will allow for the design and development of MEMS devices that could better face the necessities of future products [197]. Examples of such materials include shape-memory alloy, magnetic, magnetostrictive, electrostrictive, and piezoelectric thin films and nanomaterials, with a special focus on silicon carbide and carbon-based materials, such as diamond or graphene [199]. BioMEMS and LoCs are now mostly based on polymers as primary materials, which are widely biocompatible, but biodegradable [197]. Moreover, owing to their stimuli-responsive deformation, hydrogel-based MEMS have also attracted considerable scientific interest in the field [200].

MEMS technologies also face the challenges of developing advanced manufacturing processes in order to ensure commercialization and reduce costs [201]. Thus, the last decade has witnessed the shift towards novel techniques, such as 3D printing. The 3D printing process has the potential to eliminate the limitations of conventional lithographic manufacturing in terms of undesired underetching or overetching of 3D structures or the misalignment of the etching pattern. However, there are still considerable limitations in this area, mostly related to the lack of standardization in terms of size and the thermal shrinkage of materials in the 3D printing or post-sintering processes [202]. Additionally, recent studies have demonstrated the potential of double-exposure, double-patterning [203], and moving lens [204] photolithography technologies for the development of MEMS devices. The current trend in the field is represented by the size reduction in MEMS to nanoelectromechanical systems (NEMS), which are even smaller, lighter, and more powerful platforms [205,206]. However, the implementation of MEMS and NEMS is still limited by the challenges imposed by the difficult and complex assembly and packaging of such devices, which are generally harmful and negatively impact their yield.

Nonetheless, the field is continuously evolving, with new horizons to pursue every day. The potential applications that are constantly being researched and evolved, such as faster and more accurate point-of-care disease diagnostic devices and internet of things (IoT) solutions for the healthcare system, are closer than ever due to the multi- and interdisciplinary education of nowadays scientists and the highly advanced infrastructure available.

## 6. Conclusions

Owing to the tremendous advancements in electronics miniaturization, MEMS devices were born as a novel class of miniaturized devices that could be used in a variety of fields, especially within the medical area. The most important directions of MEMS devices within biomedical applications include highly advanced and precise disease diagnostics and monitoring, drug delivery systems with controlled release, and new generations of microsurgery through novel and improved surgical tools. MEMS devices that are commonly utilized in these domains include micropumps, micro-reservoirs, microneedles, microvalves, and micromixers. With the advancements in microfabrication techniques and the appearance of novel and suitable materials, MEMS components could be designed and developed in a wide variety of ways in order to fulfill the desired purposes. Additionally, they have allowed for high degrees of miniaturization, which further enables their implantation within the human body in order to avoid repeated interventions, allow real-time and continuous monitoring and release, and limit the side effects of conventional treatment strategies. While MEMS devices are currently present within everyday life, the biomedical field still lacks commercialization due to limited efficiency testing. Nevertheless, these devices will slowly replace the current gold standards used in medicine.

## Figures and Tables

**Table 1:** Summary of the main types of micropumps and their characteristics.

Micropump Type	Driving Mechanism	Advantages	Disadvantages
electrostatic	electrostatic forces due to membrane bending	- driving mechanism scalability - low power consumption - fast response	- challenging design - small microactuator deflections
piezoelectric	pump chamber deformation due to piezoelectric element motion	- small size - low power consumption - no electromagnetic interference - insensitivity to the fluid viscosity	-
thermo-pneumatic	intermittent compression and expansion of an air compartment due to a cyclic use of a twosome heater and cooler	-	-
bimetallic	intermittent compression and expansion due to repeated heating and cooling of two materials with different thermal expansion coefficients	- high force generation - low operating voltage - design simplicity	- small-diaphragm deflection - unsuitability at high frequencies
shape-memory alloy	phase alteration inside two solid phases	- superelasticity	-
ionic conductive polymeric films	electric field-generated stress gradient of the ionic movement	-	- highly complex fabrication process - low reproducibility during batch manufacturing
electrochemical	reversible electrochemical reactions for gas bubble expansion and electrolysis reduction in the aqueous electrolyte solution	- large driving forces - accurate flow control - low heat generation - LoC technology compliance - low power consumption	- long response time caused by the slow recombination of the gas within the working chamber
electrohydrodynamic	interaction of electrostatic forces with ions within dielectric fluids	-	-
electrowetting	changes of the surface energy of a conductive liquid in contact with a dielectric-coated electrode when an external voltage potential is applied	- flexibility - possibility of discrete liquid volumes manipulation - increased reproducibility, mobility, and reversibility	- electrochemical interactions due to the direct contact between the liquid and the electrode at low voltage input
electro-osmotic	movement of the uncharged liquid relative to the charged microchannel surfaces under the action of an externally applied electric field	- no moving parts - possibility of using fluids with a wide range of conductivities	- not suitable for electrolytic aqueous solutions
magnetohydrodynamic	movement of electrically or weakly electrically conductive liquids by the Ampère’s force	-	-
